# Economic costs incurred by the patients with multiple sclerosis at different levels of the disease: a cross-sectional study in Northwest Iran

**DOI:** 10.1186/s12883-020-01790-5

**Published:** 2020-05-23

**Authors:** Ali Imani, Farid Gharibi, Ali Khezri, Nasrin Joudyian, Koustuv Dalal

**Affiliations:** 1 Health Economics Department, Tabriz Health Service Management Research Center, Iranian Center of Excellence in Health Management, Tabriz University of Medical Sciences, Tabriz, Iran; 2 Food Safety Research Center (salt), School of Nutrition and Food Sciences, Semnan University of Medical Sciences, Semnan, Iran; 3 School of Management and Medical Informatics, Tabriz University of Medical Sciences, Tabriz, Iran; 4 School of Management and Medical Informatics, Tabriz University of Medical Sciences, Tabriz, Iran; 5 Department of Public Health Science, School of Health Sciences, Mid Sweden University, Sweden and Higher School of Public Health, al-Farabi, Kazakh National University, Almaty, Kazakhstan

**Keywords:** Multiple sclerosis, Disease cost, Disease levels, Iran

## Abstract

**Background:**

Multiple sclerosis (MS) causes significant economic burden to the patients, families, health systems and society. This study aimed to estimate the annual economic costs incurred by patients with multiple sclerosis (pwms) at different levels of the disease.

**Method:**

This was a cross-sectional study, using the Expanded Disability Status Scale (EDSS) tool for assessing the disease level of 300 (=N) pwms in East Azerbaijan province, Iran. To estimate the cost of MS, a questionnaire with its validity and reliability (CVR 92% and CVI 87%) and pilot test (Cronbach’s alpha score 0.89) was used. The data were collected by interviewing pwms and reviewing their clinical records. Multivariate linear regression was used to assess the relationship between disease levels and incurred costs.

**Results:**

The results revealed that the mean annual cost for pwms in Iran is 97,521,740 IRR (equivalent to 2321.94 USD; 1978.93 EURO) and the mean score of EDSS in pwms was 3.14. The annual cost incurred by pwms with mild, moderate and severe levels of disease were 83,918,150 IRR (1998.05 USD; 1702.88EURO), 137,772,660 IRR (3280.30 USD; 2795.71 EURO) and 119,962,670 IRR (2856.25 USD;2434.30 EURO), respectively. Also, on average, each increase in EDSS score in pwms in Iran led to increase 8,139,260 IRR (equivalent to 193.79 USD; and 165.16 EURO) in total annual cost which must paid from pwms and their households exclusively. Also, there was a significant relationship between total annual cost and disease severity in such a way that any increase in EDSS degree is led to 8,139,260 IRR (193.79 USD; 165.16 EURO) added cost for pwms.

**Conclusion:**

The study results could be helpful for Iranian health managers to solve problems which are facing by the patients with multiple sclerosis and their families.

## Background

Multiple sclerosis (MS) is a complex inflammatory disease associated with the central nervous system, resulting in several physical health problems including muscle stiffness, diplopia, sensory loss, limb weakness, gait ataxia, loss of bladder control, epilepsy, functional impairment and disability [1]. MS leads to a wide range of psychological disorders, including depression, disappointment, cognitive impairment, loss of independence, pain, fatigue, anxiety, lack of confidence, and social problems [2]. It causes significant economic burden to patients with multiple sclerosis (pwms) and societies [1, 3–5]. A study has shown that during 2016, worldwide 2.22 million people were suffering from MS with an increase of prevalence of 10.4% during the last 26 years [1]. MS has emerged as a major public health problem with its noteworthy prevalence [1, 6], long survival time [7], affecting the high productivity age [8], resulting in devastating socioeconomic effects [1, 9, 10].

Besides its physical, psychological and social effects, MS incurs huge economic costs for the pwms and their families and health systems [11]. Depending on the duration of the disease, the level of nEUROo-degeneration MS results with various level of productivity loss including unemployment [12].With a mean affecting age of MS around 30 years, studies indicated that even the working persons have lost their jobs after five to 10 years of diagnosing MS resulting severe burden of unemployment [1, 13].The prevalence of MS in Iran varies between different geographical locations [14]. There are 5–13 cases per 100,000 population in southeast Iran [15] and 33.5–51.9 cases per 100,000 population in central Iran [16]. Literatures indicate that total number of people with MS is increasing in Iran like other Middle Eastern countries [14, 17], and developed countries too [18].

Studying the disease status in pwms and the costs incurred by them is one of the essential requirements for managing this disease. There is a dearth of materials in low and middle-income countries, especially focusing on economic costs [1, 19–21]. With 304 death and 26,395 DALYs lost due to MS during 2016, Iran has a lack of health economics studies estimating the cost of MS [1]. The current study aimed to investigate the annual economic costs incurred by pwms at different levels of the disease in Iran.

## Methods

### Participants

This was a cross-sectional study, conducted in May 2018 with the participation of 300 registered pwmsin East Azerbaijan province of Iran. A simple random sampling method was used in this study and the sample size was determined based on the total number of pwms registered in this association (1200) and using the Morgan table [22]. The inclusion criteria for pwms were their registration and having medical records in the East Azerbaijan MS Association and spending at least 1 year from the diagnosis and initiating treatment. The inclusion criteria were the inability and the unwillingness of pwms for study participation. The participation rate was 97%. Nine pwms had an inappropriate health condition and could not agree to participate in the study (3% decline rate).

### Study tools

The Expanded Disability Status Scale (EDSS) questionnaire was used for calculating the clinical status of pwms [23, 24].For economic cost estimation, a cost questionnaire including demographic and related variables were designed and standardized during the study. In the first step of developing the instrument for evaluating the cost of MS, comprehensive literature reviews were conducted. Content validity and face validity of the questions were examined by 10 field experts in terms of five aspects of necessity, relevance, transparency, simplicity, and feasibility of measurement. Then, content validity ratio (CVR) was evaluated based on necessity scores. After confirming the questions in CVR index, the mean score of four other indicators or content validity index (CVI) was reviewed and confirmed. The acceptance score of 70% was selected as the decision criterion according to the responses of 10 experts [25].In this study, the CVR score (92%) and CVI score (87%) were obtained. The reliability of the questionnaire was also confirmed by the data obtained from a sample of 50 pwms in a pilot study and the Cronbach’s alpha score (α) of 0.89.

### Data collection

A pre-tested questionnaire was used for interviewing the pwms for estimating costs along with reviewing their medical records. The pwms/family (household) perspective was used to calculate the costs of the disease, which included a set of direct and indirect costs incurred by pwms and their families [26, 27]. The reference year was May 2017 to May 2018 for cost estimation. Given that the pwms had received their required healthcare service in determined healthcare facilities (clinicians, pharmacy, hospitals and rehabilitation centers), their health records were assessed precisely for assessing more accurate data. Direct costs are consisting of direct medical costs including diagnosis, treatment and rehabilitation costs; and non-medical costs including supportive equipment costs (wheelchair), equipping the home with needed medical facilities, regular transfer costs to/from care centres [28, 29]. Indirect costs refer to the lost time and related productivity loss in pwms. The indirect medical costs were calculated based on the reduction of monthly or annual income of pwms and their family members due to job loss or absence from job [30, 31]. In absence of any formal record keeping system, there is a lack of relevant clinical and managerial information for pwms in Iran, especially for non-medical and indirect cost elements. Multiple sources were used for the necessary information generation. For example, for employed pwms we had used their medical and/or job records. For self-employed pwms we had used self-reported information. In addition, indirect costs for unemployed pwms including housewives and pensioners were calculated based on the incurred cost to households due to their MS related disability. For example, the disability of housewives due to MS were led to more purchasing of services and facilities such as purchasing food from outdoor, hiring household servants.

### Data analysis

The disease status score was calculated by the EDSS questionnaire and a nEUROological examination by the nEUROologist, during which the condition and grade of disease progression in pwms. The EDSS is a self-assessed tool to determine the staus of pwms, which uses a description of disease severity focusing on ambulation. Although this is a self-assessed tool that answered from pwms, the research team was used from nurologists to enhance the delivered answers from pwms. The pwms status determined on a scale between 0 and 10; these numbers represent the best and the worst possible conditions for the pwms, respectively. Although the disease severity was categorised in three levels: mild (EDSS score less than 4), moderate (EDSS score between 4 to 6.5) and severe (EDSS score 7 and above) disability [32, 33].

Frequency, mean and standard deviation (SD) were used. The multivariate linear regression was used for assigning the relationship between the disease condition and various costs imposed on pwms. These costs are based on the exchange rates of the Central Bank of Iran, expressed in US dollars and EURO (a USD and EURO equivalent to 42,000 IRR and 49,280 IRR on 20 May 2018, respectively) [34]. Due to sanction, we could not provide international Dollar for 2018. All analyses were performed using SPSS18. Significance level was considered at *P* < 0.05.

### Ethical considerations

The study has received ethical permission from the Ethics Committee of Tabriz University of Medical Sciences (IR.TBZMED.REC.1396.101). Each participant was informed and explained by the researchers about the study objectives. Freedom of participation, privacy, anonymity, and rights to withdraw during study were explained and assured. Written informed consent was obtained from each participant.

## Results

### Characteristics of the patients with multiple sclerosis (pwms)

The mean age of the pwms was 37.15 (±9.68) years old. The youngest and oldest pwms were 16 and 68 years old, respectively. Most of the pwms were middle-aged and more than two-thirds of them were women. The mean age of the onset of the symptoms was 27.18 (±7.64) years and on average, 9.95 (± 7.06) years were passed since the onset of symptoms. These pwms were mostly married, housekeeper, native to the city of Tabriz, and with low education level. All pwms had basic insurance and the majority of them were covered by social welfare, but only one-third of the pwms had a supplementary insurance. (Table 1).

**Table 1:** Demographic and background characteristics

Variable	Category	Frequency	Percentage
Age	Childhood and adolescence (under 20 years)	14	4.67
	Youth (20–35 years)	128	42.67
	Middle age (35–60 years)	156	52.00
	Elderly (over 60 years)	2	0.66
Gender	Male	96	32.00
	Female	204	68.00
Mean age at first symptoms	Under 20 years	67	22.33
	20–29 years	122	40.67
	30–39 years	92	30.67
	40–50 years	19	6.33
Years of illness	1–5 years	96	32.00
	6–10 years	90	30.00
	11–15 years	53	17.67
	More than 15 years	61	20.33
Marriage status	Single	99	33.00
	Married	188	62.67
	Divorced and spouse died	13	4.33
Education	Illiterate	15	5.00
	Diploma and lower	157	52.33
	B.Sc.^a^	103	34.33
	M.Sc.^b^	20	6.67
	Ph.D.^c^	5	1.67
Employment	Employed (public or private sector)	27	9.00
	Self employed	10	3.33
	Student	22	7.33
	Housewife	152	50.67
	Retired	11	3.67
	Unemployed	24	8.00
	Other	54	18.00
Basic medical insurance		300	100
Insurance type	Social Welfare (TamineEjtemaei)	193	64.34
	Health Services (Khadamatdarmani)	73	24.33
	Other (Armed Forces, Banks, etc.)	34	11.33
Supplementary medical insurance		105	35
Locality	Tabriz	254	84.37
	Other cities	46	15.33

^a^Bachelor of Sciences

^b^Master of Sciences

^c^Philosephy Doctor

A nEUROologist had examined almost all of the pwms during the past year while other specialists had examined only 1 % of the pwms. A large number of pwms (73%) had brain MRI. Among the rehabilitation care services, physiotherapy was the only service that was received by the pwms. Betaferon, Diphosel and CinnoVex were the highest intake medicines for the pwms, respectively. Only 26% of the pwms used drug supplements. Results showed that 42% of the pwms were hospitalized in the past year, and about 5% of them had made some unofficial payments (bribery) to the health care staff (Table 2).

**Table 2:** Status of receiving medical care by pwms related to direct medical costs

Service type	Category	pwmsuse of resources		Frequency of use	
		Number of patients	Percentage (rate)	Mean	SD^a^
Visit	Specialist visit	294	98.00	7.11	3.77
Diagnosis	Laboratory	214	71.33	2.02	2.36
	MRI^b^	219	73.00	1.01	0.86
	CT Scan^c^	20	6.66	0.07	0.26
	Other	20	6.66	0.08	0.34
Rehabilitation	Physical therapy	40	13.33	3.55	13.89
	Occupational therapy	0	0	0	0
Medicine	Avonex	6	2.00		
	Betaferron	68	22.66		
	Rebief	4	1.33		
	CinnoVex	49	16.33		
	ReciGen	12	4.00		
	Diphosel (Dimethyl fumarate)	65	21.66		
	Other MS drugs	67	22.33		
	Supplements	79	26.33		
Hospitalization	First time	126	42.00	2.94	5.62
	Second time	43	14.33	0.50	1.61
	Third time	3	1.00	0.03	0.28
Other	Informal paid	14	4.66	0.05	0.34
	Complementary/alternative therapies	8	2.66	0.08	0.37
	Home care (medical)	5	1.66	0.15	0.42

^a^Standard deviation

^b^Magnetic Resonance Imaging

^c^Computed Tomography Scan

Four percent of the pwms received non-therapeutic and home care. Also, 8% of the pwms used walking aids devices, and only 0.7% of them considered safety and comfort issues at their homes. Pwms commuted mostly for receiving care services of private clinics, and no costs were incurred for accommodation and meals at the time of visiting health centers (Table 3).

**Table 3:** Status of direct non-medical costs imposed on pwms

Service type		Patients		Frequency of receiving service	
		Frequency	Percentage	Mean	SD^a^
Home care (non-medical)		13	4.33	14.23	21.66
Walking aids devices		24	8.00	0.12	0.42
Modification of home, care and …		2	0.66	0.02	0.13
Travel	Hospital	174	58.00	24.29	22.55
	Physician office	211	70.33	48.07	28.18
	Other centers	99	33.00	7.23	13.24
Accommodation and food		0	0	0	0

^a^Standard deviation

Most of the pwms (84%) and their caregivers (77%) had to be absent from work due to the disease and receiving medical care. Accordingly, pwms and their families lost 32 and 5 working days in a year on average, respectively and 15% of the pwms and 1% of their families lost their jobs due to the disease.

Investigating the amount of annual costs imposed on pwms in Irans howed that the highest proportion of the costs is related to direct medical costs and the lowest part to indirect costs. On average, the pwms paid 70,106,490 IRR (1669.20 USD; 1422.61EURO) in a year for medical/clinical services, with the cost of medicine, rehabilitation services and diagnostic services having the highest shares, respectively. The amount of direct non-medical costs for pwms was 22,201,750 IRR (528.61 USD; 450.52EURO); the highest cost was related to commuting to care centers. These results also estimated the indirect costs of pwms to be 5,213,500 IRR (124.13 USD; 105.79 EURO); these costs were mainly due to the pwmsabsence from his/her job for medical and treatment reasons. In total, the annual mean amount of 97,521,740 IRR (2321.94 USD; 1978.93EURO) was the cost for the pwms in Iran annually (Table 4).

**Table 4:** The overall costs incurred by pwmsunder study

Cost type	Category	Cost amount (IRR^a^)		Cost amount (USD^b^)		Cost amount (EURO^c^)	
		Mean	SD^d^	Mean	SD	Mean	SD
Medical Direct Costs	Physician visit	2,519,500	1,745,300	59.97	41.55	51.12	35.41
	Diagnosis	4,727,000	5,804,350	112.54	138.19	95.92	117.78
	Rehabilitation	9,481,830	99,625,340	225.75	2372.03	192.40	2021.61
	Medicine	50,757,550	61,977,004	1208.51	1475.64	1029.988	1257.65
	Hospitalization	2,010,200	4,062,930	47.86	96.73	40.79	82.44
	Home care (medical)	328,660	4,866,630	7.82	115.87	6.66	98.75
	Complementary/alternative therapies	163,330	1,267,360	3.88	30.17	3.31	25.71
	Informal paid	118,400	629,500	2.81	14.98	2.40	12.77
	Subtotal	70,106,490	121,767,380	1669.20	2899.22	1422.61	2470.92
Direct Non-Medical Costs	Home care (non-medical)	2,812,010	24,696,900	66.95	588.02	57.06	501.15
	Walking aids devices	5,354,330	58,966,640	127.48	1403.96	108.65	1196.56
	Modification of home, care and …	216,660	2,657,050	5.15	63.26	4.39	53.91
	Travel to healthcare centers	13,818,730	13,110,770	329.01	312.16	280.41	266.04
	Subtotal	22,201,750	73,513,150	528.61	1750.31	450.52	1491.74
Indirect Costs	Patients’ absence cost	4,398,870	22,167,790	104.73	528.80	89.26	449.83
	Relatives’ absence cost	2,618,160	8,388,760	62.33	199.73	53.12	170.22
	Subtotal	5,213,500	18,987,350	124.13	452.07	105.79	385.29
**Total**		97,521,740	145,680,370	2321.94	3468.58	1978.93	2956.17

^a^The Iranian Rial

^b^United States Dollar

^c^The EUROope Currency

^d^Standard deviation

Reviewing the status disease in pwms indicated that the mean score of EDSS was 3.14. Also, 70% of the pwms had mild illness and each group of the pwms with moderate and severe disease comprised about 15% of the total. Nearly one-third of the pwms had suffered a recurrence of the disease during the past year, and the mean recurrence rate for this period was 0.58 times (Table 5).

**Table 5:** Status of the disease in the pwms under study

Variable	Category	Frequency	Percentage
EDSS score	1	139	46.33
	1.5	1	0.33
	2	29	9.67
	3	13	4.33
	3.5	1	0.33
	4	27	9.00
	4.5	2	0.67
	5	20	6.67
	5.5	3	1.00
	6	21	7.00
	6.5	1	0.33
	7	17	5.67
	7.5	1	0.33
	8	17	5.67
	9	5	1.67
	10	3	1.00
Disease severity	Mild disease	210	70
	Moderate disease	47	15.67
	Severe disease	43	14.33
Relapses during last year	Yes	93	31.31
	No	204	68.69
Relapses during last 3 months	Yes	85	28.91
	No	209	71.09

Regression analysis, which was adjusted for the available potential co-variables, showed that there is a significant relationship between disease status in pwms and direct medical costs and total cost. Degree of increase in EDSS score (worsening of pwms condition), enhance the direct medical costs and the total annual cost increase significantly as 7,462,080 and 8,139,260 IRR, respectively (equivalent to 177.66 and 193.79 USD; 151.42 and 165.16 EURO). The relationship between EDSS score and direct non-medical costs and indirect costs was not significant (Table 6). B indicates occurring changes in various types of cost due to one unit change in EDSS score.

**Table 6:** The relationship between EDSS score and various costs imposed on pwms

Cost type	IRR^a^		USD^b^		EURO^c^		β^e^	***P***-value
	B^d^	Std. Error	B^d^	Std. Error	B^d^	Std. Error		
Direct medical costs	7,462,080	27,233,210	177.66	648.40	151.42	552.62	0.157	0.007
Direct non-medical cost	1,426,870	1,662,640	33.97	39.58	28.95	33.73	0.050	0.391
Indirect cost	− 749,700	427,760	−17.85	10.18	−15.21	8.68	−0.101	0.081
Total cost	8,139,260	3,265,050	193.79	77.73	165.16	66.25	0.143	0.013

^a^The Iranian Rial

^b^United States Dollar

^c^The EUROope Currency

^d^B or unstandardized beta: it refers to the slope of the line between the predictor variable (EDSS score) and the dependent variable (costs of illness)

^e^β or standardized beta: it refers to the percentage change in dependent variable (costs of illness) due to a unit change in predictor variable (EDSS score)

The results also indicated that pwms in Iran with mild illness spent 83,918,150 IRR (1998.05USD; 1702.88 EURO); pwms with moderate illness spent 137,772,660 IRR (3280.30 USD; 2795.71EURO); and the cost for pwms with severe illness was 119,962,670 IRR (2856.25 USD; 2434.30EURO) annually. Also, the regression model which was done to determine the statistical relationship between the disease condition (mild, moderate and severe) and illness costs, it was found that the status of pwms had a significant relationship with direct medical costs and the total cost (Table 7).

**Table 7:** The relationship between status of pwmsand types of costs imposed on them

Cost type	Mild disease				Moderate disease				Severe disease				***P***-value
	Mean (SD^a^)			% of total cost	Mean (SD)			% of total cost	Mean (SD)			% of total cost	
	IRR^b^	USD^c^	EURO^d^		IRR	USD	EURO		IRR	USD	EURO		
**Direct medical costs**	57,330,010 (±61,057,540)	1365.00 (±1453.75)	1163.35 (±1238.99)	68.31	111,768,420 (±255,325,660)	2661.16 (±6079.18)	2268.02 (±5181.12)	81.12	86,965,810 (±112,051,120)	2070.61 (±2667.88)	1764.72 (±2273.77)	72.49	0.013
**Direct non-medical cost**	19,949,800 (±80,598,510)	474.99 (±1919.01)	404.82 (±1635.52)	23.78	25,227,650 (±57,750,230)	600.65 (±1375.00)	511.92 (±1171.87)	18.23	29,892,200 (±48,967,780)	711.71 (±1165.89)	606.57 (±993.66)	24.92	0.690
**Indirect cost**	6,638,330 (±22,281,580)	158.06 (±530.27)	134.70 (±452.14)	7.91	776,590 (±3,544,450)	18.49 (±84.39)	15.75 (±71.92)	0.56	3,104,650 (±6,910,610)	73.92 (±164.53)	63.00 (±140.23)	2.59	0.118
**Total cost**	83,918,150 (±10,316,453)	1998.05 (±245.62)	1702.88 (±209.34)	100	137,772,660 (±264,022,250)	3280.30 (±6268.24)	2795.71 (±5357.59)	100	119,962,670 (±136,103,800)	2856.25 (±3240.56)	2434.30 (±2761.84)	100	0.039

^a^Standard deviation

^b^The Iranian Rial

^c^United States Dollar

^d^The EUROope Currency

## Discussion

The study was performed to determine the costs of pwms in different levels of disease in Iran. The results can help the health managers and policy makers in developing fair health care delivery to the pwms using an evidence-based and effective support system. The assessed characteristics of pwms in the current study showed that majority of them (68%) were women (with mean age 37.15 years); which is in line with a Norwegian study [3], where, 65.1% pwms were women (mean age 37.7 years) and a Swedish study [24], where 73% of participants were women (mean age 53.4 years). Middle-aged unemployed women are mostly affected by MS. The pwms in Iran has more (42.7%) university education than Swedish (25.8%) pwms [24]. The majority of the pwms were not employed in the current study which is supported by earlier studies from Scandinavian countries [3, 24].

The study results indicated that, on an average, pwms in Iran spent an annual amount of 97,521,740 IRR (2321.94 USD; 1978.93 EURO). Also, on average, each increase in EDSS score in pwms in Iran led to increasing 8,139,260 IRR (equivalent to 193.79 USD; and 165.16 EURO) in total annual cost which must be paid by the pwms and their households exclusively. The highest share of expenses (almost 72% of total cost) was related to direct medical costs and the lowest share (5%) to indirect costs. In a study by Imani et al. (2013) in Iran, the mean annual total cost per pwms, in a societal perspective, was estimated to be 24,475 USD, 20,859 EURO and direct medical costs accounted for the largest share of the costs too [35]. In the studies by Svendsen et al. (2012) in Norway and Berg et al. (2006) in Sweden, the annual cost of the disease was estimated to be 65,000 and 53,600 EURO, respectively [3, 24]. The direct medical cost, direct non-medical cost, and indirect cost were 71.89, 22.77 and 5.34% respectively in this study, in Iran. While the direct medical cost, direct non-medical cast, and indirect cost were 22.45, 16.59 and 60.69% respectively in Norway [3]; and were 46.91, 21.10 and 31.99% respectively in Sweden [24]. The pattern of incurred cost in Iran and Sweden is the same. In Iran the direct medical costs are constituting the major cost part, while in Sweden the indirect costs is more than direct non-medical costs. Although in Norway, the direct medical costs are more than direct non-medical costs but the indirect costs have more share in total annual costs than other type of costs. The cost of disease in different countries with varying contexts could not make comparable as the health systems, healthcare provider organizations, health status, structures, and mechanisms for payments, as well as healthcare utilization and the caregivers’ perspectives are different [24, 36]. In addition, the study perspective can affect these results because the conducted studies in Norway and Sweden have societal perspective, while the current study has household perspective. It is important to consider issues such as low and declining value of Iranian national currency, the economic sanctions and, consequently, the scarcity of drugs, and high prices of the drugs due to the lack of manufactures in Iran.

Reviewing the conditions of MS suggested that the average score of EDSS in pwms was 3.14, and about 70% of them were in the mild group, and each of the groups of pwms with moderate and severe illness comprised 15% of the sample. In Sweden, the mean EDSS score was 5.1, and the mild, moderate and severe levels of disease included 29, 46, and 25% of the total pwms, respectively [24].In Norway, the mean score of EDSS was 4.3, and the mild, moderate and severe levels of the disease included 43.5, 43 and 13.5% of total pwms, respectively [3].The reason for the better status of the disease in this study may be attributed to early diagnosis and treatment because the mean age of the pwms at the time of diagnosis in Sweden was 39 and it was 37 in Norway. But in the present study, the mean age of the pwms was 27 years. This has led to the fact that, despite 10 years since the onset of the disease, pwms are still in mild condition in Iran. Undoubtedly, part of this difference in the condition of illness can be attributed to the limitation of the present research, which could not access pwms in severe condition because of their reluctance to participate in the study.

The current study supports the literature that there is a significant correlation between the severity of the disease (EDSS score) and various types of costs imposed on pwms, so that more EDSS led to more annual cost to pwms [24]. The annual cost incurred by pwms in Iran with mild, moderate and severe levels of MS disease were 83,918,150 IRR (1998.05 USD; 1702.88 EURO), 137,772,660 IRR (3280.30 USD; 2795.71 EURO) and 119,962,670 IRR (2856.25 USD; 2434.30 EURO) respectively. Another study demonstrated that the mean annual costs in mild, moderate and severe disease in Europeans were 22,800 EURO, 37,100 EURO and 57,500 EURO, respectively [32]. The conducted study in Italy also declared that the mean annual costs in mild, moderate and severe disease in Italian people were 22,750 EURO, 43,616 EURO, 63,047 EURO, respectively [33]. The annual costs in those studies were more because they used societal perspective, while the current study had used household perspective. The current study could help the policy makers to better plan the health systems in the low- and middle-income countries. In a fairly established health system, the majority of costs are paid by health system and insurance foundations. In the low- and middle-income countries big amounts of the costs were paid the households.

The reason of increasing occurred costs on pwms by worsening EDSS score could be seen in the insurance systems, social security and poor financial support for pwms in Iran than in high-income countries [10, 36]. In this situation, providing proper insurance system and necessary social security which cover all of required services with reasonable and payable cost for pwms and their families (Universal Health Coverage / effective coverage) could lead to proper financial protection and healthcare access. Also, costly care increases concern of pwms on direct medical costs (as the major cost) and obliges them to neglect other indirect costs associated with the disease. For example, a recent study from Germany demonstrated that healthcare costs increases with severity but the in-patients with MS costs declined due to specific treatment [34].

Comparison of the amount of costs imposed on pwms at different levels of EDSS score showed that direct medical costs in pwms with severe disease were higher than those with mild illness and lower than those with moderate type of disease. The reason for high direct medical costs imposed on pwms with severe condition is that they need more diagnostic, therapeutic and rehabilitation facilities. Moreover, the lower costs of disease in pwms with severe conditions compared to moderate pwms can be associated with bad economic conditions of the first group due to a high amount of total cost as well as a decrease in their earnings because of absence from work and losing their jobs. All these factors make the pwms with severe disease take cheaper drugs with low efficiency or put some essential treatment services aside resulting decrease in productivity and extreme situation unemployment. This might be another reason why the severe pwms were unwilling to participate in the current study.

The lack of similar studies which assess disease costs from the household perspective (analysing incurred cost by the pwms and their families, not the incurred cost by the health systems or society) in Iran or other developing countries especially in Eastern Mediterranean Region is noteworthy. The research propose some executive suggestions, including: strengthening the basic and supplementary insurance system for pwms, strengthening their social security system, increasing governmental and charitable support, enhancing social work for pwms, in particular, creating jobs and income generation for pwms in proportion to their physical capacity, establishing comprehensive caring centers for pwms to provide one-stop-shop type of services for all necessary cares, providing full coverage of pwms cares by insurers, and providing government planning to ensure timely delivery of medicines and equipment to healthcare managers in Iran. Further studies in Iran and other low- and middle-income countries are essential for estimating a national level cost of illness of MS. Also, to assess the share of MS costs in the household budget (% of average income spent on healthcare expenses or MS-related expenses) to estimate the scale and level of catastrophic expenditure and its induced impact on poverty in the pwms and their families are warranted.

The current study is a cost of illness study which may have some criticism [10, 27, 36].However, it can provide important information as direct costs being the main cost driver, to ensue with marginal analyses which in turn help inform priority setting for MS for scarce resource allocation in economically struggling Iran by decision-makers.

## Conclusions

The study results showed that by increasing the severity of illness (EDSS score) in pwms, higher costs are incurred by them and their families especially due to their increasing need to receive more and costly medical services. The amount of costs imposed on pwms in Iran was very high and is increasing, while their income is decreasing. This study also showed that the health systems and medical insurance systems have not enough financial support and have not protected from pwms, particularly be disease progression, because only one-third of pwms have supplementary medical insurance. The results of this study can pave the way for Iranian healthcare managers and policymakers to provide financial, social and psychological support to pwms and their families.

## Data Availability

The study data are available and will send to made accessible by Dr. Farid Gharibi (Email: gharibihsa@gmail.com). Persian questionnaires are appended in appendix.

## Abbreviations

CVI: Content validity index
CVR: Content validity ratio
EDS: Expanded disability status scale
MS: Multiple sclerosis
PWMS: patients with multiple sclerosis
